# Prevalence and characterization of ESBL-producing *Escherichia coli* in healthy pregnant women and hospital environments in Benin: an approach based on Tricycle

**DOI:** 10.3389/fpubh.2023.1227000

**Published:** 2023-09-28

**Authors:** Kevin Sintondji, Kafayath Fabiyi, Jules Hougbenou, Hornel Koudokpon, Boris Lègba, Hornella Amoussou, Kaisa Haukka, Victorien Dougnon

**Affiliations:** ^1^Research Unit in Applied Microbiology and Pharmacology of Natural Substances, Research Laboratory in Applied Biology, Polytechnic School of Abomey-Calavi, University of Abomey-Calavi, Cotonou, Benin; ^2^Department of Microbiology, University of Helsinki, Helsinki, Finland

**Keywords:** ESBL-producing *Escherichia coli*, pregnant women, hospital settings, Tricycle protocol, Benin

## Abstract

**Introduction:**

Extended-Spectrum Beta-Lactamase (ESBL)-producing *Enterobacterales* are recognized as significant pathogens due to their resistance to multiple antibiotics. This study aimed to determine the prevalence of ESBL-producing *Escherichia coli* (*E. coli*) in different settings, including healthy pregnant women, the food chain, and the environment of tertiary hospitals in Benin.

**Methods:**

Samples were collected from various sources, including fecal samples from healthy pregnant women, food samples from hospital canteens, and hospital effluents from four tertiary hospitals in southern Benin. Fecal samples were plated on MacConkey agar supplemented with cefotaxime (4 μg/mL), while food and water samples were plated on Tryptone Bile X agar supplemented with cefotaxime (4 μg/mL). Urea indole tests were used for preliminary identification of *E. coli* colonies, followed by confirmation of ESBL production using the double disk synergy technique. Antibiotic susceptibility testing of ESBL-producing *E. coli* strains was conducted using the disk diffusion method on MH agar. Polymerase Chain Reaction (PCR) was used to investigate the presence of ESBL-encoding genes.

**Results:**

Among the 296 fecal samples collected from four tertiary hospitals, ESBL-producing *E. coli* was isolated from 22.30% (66) of the samples. All *E. coli* isolates from hospital effluents exhibited ESBL production, while ESBL-producing *E. coli* was not detected in food and drinking water samples. The analysis of variable associations showed no significant associations (*p* > 0.05) for the studied factors. Antibiotic susceptibility testing revealed high resistance rates among the ESBL-*Ec* isolates against several tested antibiotics, including amoxicillin, aztreonam, ceftriaxone, ciprofloxacin, and trimethoprim-sulfamethoxazole. However, most isolates remained susceptible to ertapenem, amoxicillin-clavulanate, and imipenem. The most prevalent ESBL-encoding genes were *bla_TEM_* (37.50%), *bla_OXA-1_* (19.44%), and *bla_SHV_* (11.11%), while a smaller proportion of isolates carried *bla_CTXM-1_*/*bla_CTXM-15_* (5.55%) and *bla_CTXM-9_*.

**Discussion:**

This study provides insights into the prevalence of ESBL-producing *E. coli* carriage in the feces of healthy pregnant women in southern Benin. Additionally, it highlights hospital wastewater as a potential reservoir of ESBL-producing bacteria in the environment. The detection of ESBL-producing *E. coli* in hospital effluents raises concerns about the dissemination of antibiotic resistance genes into the environment. The high resistance rates observed among ESBL-*Ec* isolates against commonly used antibiotics emphasize the urgent need for antimicrobial stewardship and infection control measures. The identification of prevalent ESBL-encoding genes contributes to understanding the genetic basis of ESBL resistance in the studied population. Further research is warranted to explore the mechanisms of transmission and potential interventions to mitigate the spread of ESBL-producing Enterobacterales.

## 1. Introduction

The emergence and spread of antibiotic resistance pose a significant global threat to public health, affecting humans, animals, and the environment (1). Of particular concern is the rise in bacteria resistant to multiple drugs, including Extended-Spectrum Beta-Lactamase Producing *Enterobacterales* (ESBL-PE) (2). While traditionally associated with hospital settings, there is a growing incidence of community-acquired ESBL-PE infections (3). The prevalence of ESBL-producing *Enterobacterales* has been steadily increasing, especially in middle-and low-income countries, since the early 2000s (4, 5). In Africa, approximately 20% of healthy individuals have been reported to carry ESBL-PE, according to a meta-analysis by Karanika et al. (6). This situation has significant consequences, including the limitation of treatment options, the occurrence of severe complications, prolonged hospitalization, and increased healthcare costs (7). Consequently, combating antibiotic resistance has become a global priority.

In response to the rapid emergence and dissemination of resistant bacteria and genes, integrated surveillance systems have been established to address antibiotic resistance as a One Health concern (8). One such surveillance protocol is the Tricycle approach proposed by the World Health Organization (9). Tricycle aims to monitor the prevalence of ESBL-*E. coli* (ESBL-*Ec*), a key indicator, in three primary domains: humans, the food chain, and the environment. *E. coli* is a suitable focus for surveillance due to its ubiquitous presence in the environment and its commensal nature in the human gut (10). ESBL-*Ec*, known for its resistance to critically important antimicrobials, serves as a significant indicator for assessing the global antimicrobial resistance (AMR) issue (5). Infections caused by ESBL-*Ec* result in severe illnesses and fatalities, imposing substantial burdens on healthcare systems (11). Since the prevalence of ESBL-*Ec* in human fecal carriage is higher in the hospital than in the community settings (12, 13), the hospital environment serves as a reservoir and transmission hub for pathogens (14, 15). The characteristics of healthcare facilities in developing countries, including high population density and extensive antibiotic use, contribute to the emergence and spread of antibiotic-resistant strains (16). In the Tricycle protocol, the human sector includes healthy pregnant women as representatives of the community (5). The carriage of ESBL-*Ec* during pregnancy raises concerns about the transmission of resistant strains to infants and the increased risk of transmission within healthcare settings. Additionally, the hospital environment, including food services and water sources, can be a potential source of contamination that contributes to longer hospital stays and hospital-acquired infections (17, 18). Furthermore, resistant bacteria, such as ESBL-producing *E. coli*, can be disseminated to the community and farms through hospital effluents and sewage treatment plants (19).

Although studies have reported the presence of ESBL-producing bacteria and their resistance genes in various samples from Africa and globally (7, 20–23), limited data exist on the prevalence of ESBL-producing *Enterobacterales* in healthy pregnant women, the food chain, and the environment in Benin (21, 24–28). Therefore, the present study investigates the prevalence and characteristics of ESBL-producing *E. coli* in healthy pregnant women, the hospital environment, and the food chain within various tertiary hospitals in Benin. By shedding light on the extent of ESBL-*Ec* carriage in these settings, this research will contribute to the understanding of antibiotic resistance patterns and guide effective infection control measures and treatment strategies.

## 2. Materials and methods

### 2.1. Study design

This study was a cross-sectional and descriptive study conducted from January to February 2023 across four tertiary hospitals located in southern Benin. The study population consisted of healthy pregnant women attending antenatal care at the target hospitals. Pregnant women who were ill or hospitalized, refused to give informed consent, or were unable to provide a stool sample were excluded from the study. Socio-demographic data were collected along with the stool samples. The sampling and microbiological methods followed the Tricycle protocol, with modifications made to include food sample analysis and adapt to the scale of the hospital. The Ethics and Research Committee of the Institute of Applied Biomedical Sciences (CER-ISBA) reviewed and granted approval to the research proposal with the reference number 154. Prior to participating in the study, every patient or their parent/guardian provided written informed consent and received a brief explanation regarding the study’s purpose.

### 2.2. Sample collection

The sample size for fecal samples was determined using the Schwartz formula (29), $n=\frac{z^{2}\times p\times q}{d^{2}}$ where: n = required sample size, p = prevalence; p = 0.21 (30); q = 1-p; z = confidence level based on the centered normal distribution (for a 95% confidence level, z = 1.96); d = tolerated margin of error for this study, equal to 0.05. A minimal sample size of 255 was calculated. A total of 335 samples including 296 fecal samples, 15 foods samples, 12 drinking water samples and 12 hospital effluents were collected during the study period. Fresh stools were collected in sterile containers, and rectal swabbing was performed for patients who had difficulty defecating but consented to participate. For other sample types, approximately 200 g of cooked meal was purchased from vendors under sales conditions and transferred into sterile sampling bags. Drinking water and hospital effluent samples were collected in sterile 500 mL bottles. All collected samples were placed in coolers with cold blocks. A questionnaire was administered to collect data on age, pregnancy’s stage, living area, educational level, access to basic amenities, presence of pets or farm animals, and recent antibiotic use.

### 2.3. Microbiological analysis

#### 2.3.1. Detection of ESBL-producing *E. coli*

Guidelines set by the World Health Organization (WHO) on integrated global surveillance of ESBL-producing *E. coli* were followed when performing the isolation and identification processes (9). Stool samples were plated on MacConkey agar supplemented with cefotaxime 4 μg/mL for the presumptive detection of ESBL-producing *E. coli*. Decimal dilutions of food samples were done in peptone water and the dilutions were inoculated onto Tryptone Bile X agar supplemented with 4 μg/mL of cefotaxime for the presumptive detection of ESBL-producing *E. coli*. Water samples were filtered through a 0.45 μm membrane, and the membranes were placed on Tryptone Bile X agar supplemented with 4 μg/mL of cefotaxime for the presumptive detection of ESBL-producing *E. coli*. Plates were incubated at 37° for 18 h, and representative colonies of *E. coli* were purified for bacterial identification through urea indole tests.

#### 2.3.2. Confirmation of ESBL-producing *E. coli* and antibiotic susceptibility test

Confirmation of ESBL production was done using the double disk synergy technique (31). Antibiotic susceptibility testing of ESBL-producing *E. coli* strains was performed using the disk diffusion method on Mueller Hinton agar according to the EUCAST recommendations (32). Various antibiotics were used, including ciprofloxacin (CIP), aztreonam (ATM), chloramphenicol (C), colistin sulfate (CS), amikacin (AK), ofloxacin (OFX), amoxicillin (AML), amoxicillin-clavulanate (AMC), sulphamethoxazole trimethoprim (SXT), and ertapenem (ETP). *E. coli* ATCC 25922 was used as a non-ESBL strain, and a clinical ESBL-producing *E. coli* was used as a positive ESBL strain.

### 2.4. Detection of resistance genes by PCR

The DNA of isolated strains showing phenotypic resistance to beta-lactams and carbapenems was extracted using “Zymo DNA Mini Kit” extraction kits according to the manufacturer’s instructions. PCR was used to detect beta-lactam resistance genes (*bla_TEM_, bla_SHV_, bla_OXA-1,_ bla_CTXM-1,_ bla_CTXM-9,_* and *bla_CTXM-15_*) and carbapenem resistance genes (*bla_KPC_, bla_NDM_, bla_IMP_, bla_VIM_,* and *bla_OXA-48_*) following the PCR conditions described by Dallenne et al. (33), Memariani et al. (34), and Cerezales et al. (35). All primer sequences are listed in Table 1. DNA fragments were analyzed by electrophoresis on a 2% agarose gel for 30 min at 100 V using BioRad Horizontal Electrophoresis System. The migration was imaged using the Biorad Gel Doc EQ imaging system, followed by interpretation of the results based on comparison of the migration of the fragments to the marker sizes (100 bp).

**Table 1:** List of the genes detected in this study.

Genes	Primers	Sequences	Annealing temperature	Product length (bp)	References
*bla*_KPC_	*bla_KPC_ F*	CGCCAATTTGTTGCTGAAGG	57°C	312	Cerezales et al. (35)
	*bla_KPC_ R*	CAGGTTCCGGTTTTGTCTCC			
*bla*_NDM_	*bla_NDM_ F*	GTTTGATCGTCAGGGATGGC	57°C	517	
	*bla_NDM_ R*	CTCATCACGATCATGCTGGC			
*bla*_VIM_	*bla_VIM_ F*	GATGGTGTTTGGTCGCATATC	58°C	202	
	*bla_VIM_ R*	CGTCATGAAAGTGCGTGGAG			
*bla*_IMP_	*bla_IMP_ F*	GAAGGCGTTTATGTTCATAC	51°C	587	
	*bla_IMP_ R*	GTACGTTTCAAGAGTGATGC			
*bla*_OXA-48_	*bla_OXA-48_ F*	GGTAGCAAAGGAATGGCAAGAA	59°C	611	
	*bla_OXA-48_ R*	CGACCCACCAGCCAATCTTA			
*bla*_GES_	*bla_GES_ F*	CTCAGATCGGTGTTGCGATC	57°C	416	
	*bla_GES_ R*	TGTATCTCTGAGGTCGCCAG			
*bla_TEM_*	*bla_TEM_ F*	CATTTCCGTGTCGCCCTTATTC	60°C	800	Dallenne et al. (33)
	*bla_TEM_ R*	CGTTCATCCATAGTTGCCTGAC			
*bla_SHV_*	*bla_SHV_ F*	AGCCGCTTGAGCAAATTAAAC	60°C	713	
	*bla_SHV_ R*	TCCCGCAGATAAATCACCAC			
*bla _OXA-1-like_*	*bla _OXA-1-like_ F*	GGCACCAGATTCAACTTTCAAG	60°C	564	
	*bla _OXA-1-like_ R*	GACCCCAAGTTTCCTGTAAGTG			
*bla_CTXM-1_*	*bla_CTXM-1_ F*	TTAGGAARTGTGCCGCTGYA^b^	60°C	688	
	*bla_CTXM-1_ R*	CGATATCGTTGGTGGTRCCAT^b^			
*bla_CTXM-9_*	*bla_CTXM-9_* F	TCAAGCCTGCCGATCTGGT	60°C	561	
	*bla_CTXM-9_* R	TGATTCTCGCCGCTGAAG			
*bla_CTXM-15_*	*bla_CTXM-15_* F	CACACGTGGAATTTAGGGACT	55°C	995	Memariani et al. (34)
	*bla_CTXM-15_* R	GCCGTCTAAGGCGATAAACA			

### 2.5. Statistical analysis

Data entry was performed using Excel 2019, and statistical analysis was conducted using R software (R version 4.0.3). Initially, a univariate analysis was performed, and variables with a *p*-value less than 0.05 were considered potentially associated with ESBL-*Ec* carriage in patients. A step-by-step analysis and assessment of interactions were conducted, and variables with a *p*-value less than 0.05 were independently considered associated with ESBL-*Ec* carriage. The results were reported as odds ratios (OR) with their corresponding 95% confidence intervals, using an interpretation threshold of α = 0.05. Additionally, the Chi-square test was performed to determine the degree of association, as logistic regression indicates a statistical link between factors and independent variables, while the Chi-square test demonstrates the level of obligation.

**Table 2:** Distribution of ESBL-producing *E. coli* per sample type.

Type of samples	Number of samples	ESBL-*Ec* positive
Stools from pregnant woman	296	66 (22.30%)
Foods	15	0
Drinking water	12	0
Hospital effluents	12	6 (50%)
Total	335	72 (21.49%)

## 3. Results

### 3.1. Prevalence of ESBL-producing *E. coli*

A total of 335 samples were collected from four tertiary hospitals and 21.49% (72) were found to be positive for ESBL-producing *E. coli*. Among fecal samples (296), ESBL-producing *E. coli* was isolated from 22.30% (66) of the samples. ESBL-producing *E. coli* was isolated from 50% (06) of hospital effluent samples. All the *E. coli* isolates obtained from hospital effluents were ESBL producers. However, no ESBL-producing *E. coli* was detected in food and drinking water samples (Table 2).

### 3.2. Risk factors associated with the carriage of ESBL-producing *E. coli*

The degree of association between factors including age, residence area, level of education, pregnancy’s stage, number of pregnancies, type of toilets, electricity access, drinking water access, pets or farm animals, immunodeficiency, recent antibiotic intake and the carriage of ESBL-producing *E. coli* was assessed. The study of the association of variables showed no association (*p* > 0.05) with the factors studied (Table 3).

**Table 3:** Factors studied for association with the carriage of ESBL-producing *E. coli*.

Variables		Number of positive ESBL-*Ec*	Univariable logistic regression	
			OR (95%)	*p*-value
Age	<21	05/09	1	0.26
	21–25	26/32	3.4 (0.68–17.54)	0.12
	26–31	16/23	1.82 (0.35–9.15)	0.45
	31–36	16/22	2.13 (0.40–11.07)	0.35
	>36	03/08	0.48 (0.06–3.27)	0.45
Living area	Urban	49/73	1	0.22
	Rural	17/21	2.08 (0.68–7.83)	0.22
Level of education	No formal education	11/13	4.58 (074–39.60)	0.11
	Elementary	06/11	1	0.28
	Secondary	33/46	2.11 (0.52–8.27)	0.27
	University	16/24	1.66 (0.37–7.32)	0.49
Pregnancy’s stage	First trimester	16/22	1	0.23
	Second trimester	35/46	1.19 (0.35–3.73)	0.76
	Third trimester	15/26	0.51 (0.14–1.69)	0.28
Number of pregnancies	No previous pregnancy	17/22	1	0.55
	1	22/33	0.58 (0.15–1.95)	0.39
	2	09/18	0.29 (0.07–1.10)	0.07
	3	09/11	1.32 (0.23–10.54)	0.76
	>3	09/10	2.64 (035–54)	0.40
Type of a toilet	Private	223	1	0.55
	Public	73	1.37 (0.49–4.21)	0.55
Electricity access	Yes	64/91	1	0.89
	No	02/03	0.84 (0.07–18.60)	0.89
Drinking water access	Yes	53/77	1	0.53
	No	13/17	1.47 (0.46–5.64)	0.53
Pets or farm animals	Yes	17/21	1	0.22
	No	49/73	0.48 (0.12–1.4)	0.22
Immunodeficiency	Yes	05/06	1	0.47
	No	61/88	0.45 (0.02–2.98)	0.47
Recent antibiotic intake	No	16/23	1	0.93
	No	50/71	1.04 (0.35–2.83)	0.93

### 3.3. Antibiotic susceptibility profiles of the ESBL-producing *E. coli* strains

**Figure 1:**
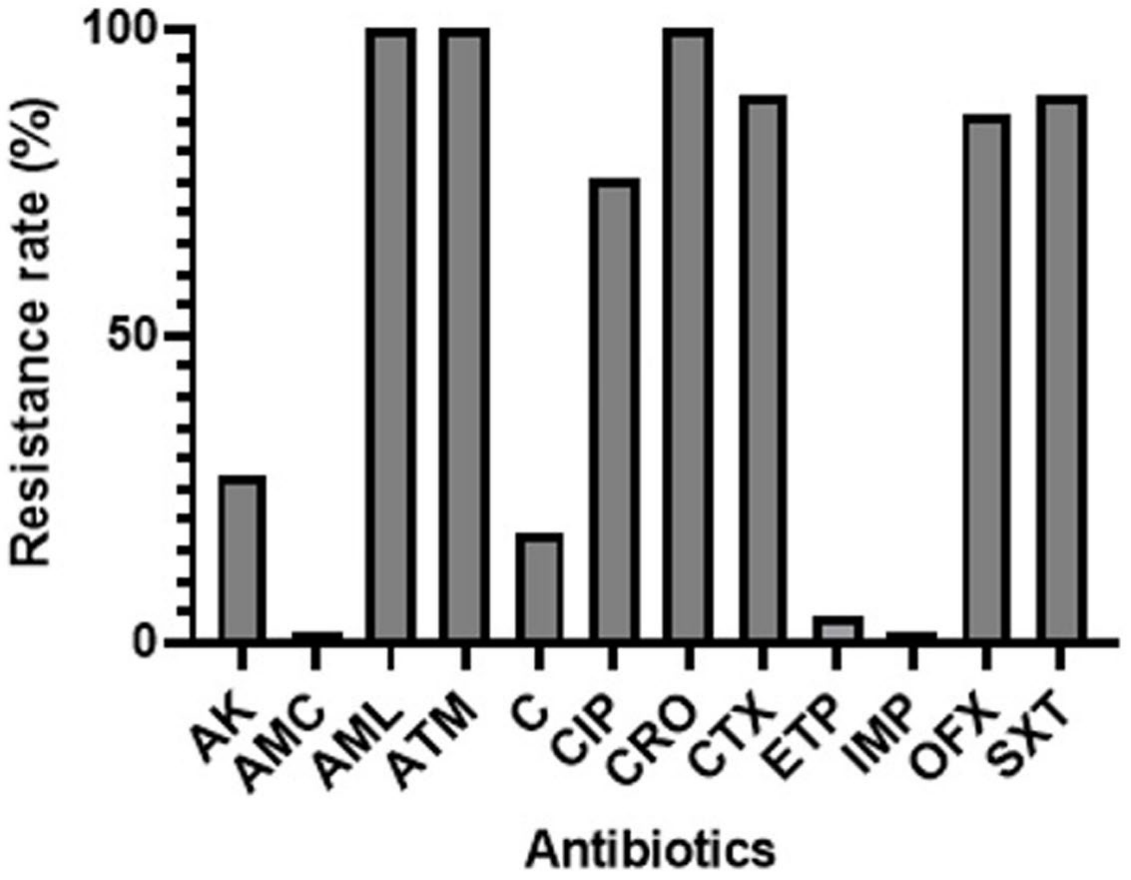
Percentage rates of antibiotic resistance among ESBL-*Ec* isolates from pregnant women. CIP, ciprofloxacin; AML, amoxicillin; ATM, aztreonam; CTX, cefotaxime; AMC, amoxicillin-clavulanate; IMP, imipenem; ERT, ertapenem; AK, amikacin; C, chloramphenicol; OFX, ofloxacin; SXT, trimethoprim-sulfamethoxazole; CRO, ceftriaxone.

The antibiotic susceptibility rates of the ESBL-producing *E. coli* isolated strains from pregnant women are presented in Figure 1. The results revealed that all the isolates (100%) exhibited resistance to amoxicillin, aztreonam, and ceftriaxone. High rate of resistance was observed for trimethoprim-sulfamethoxazole (89.39%), cefotaxime (89.39%), ofloxacin (86%), and ciprofloxacin (75.75%). However, nearly all isolates demonstrated susceptibility to ertapenem (4.54%), amoxicillin-clavulanate (1.51%), and imipenem (1.51%).

**Figure 2:**
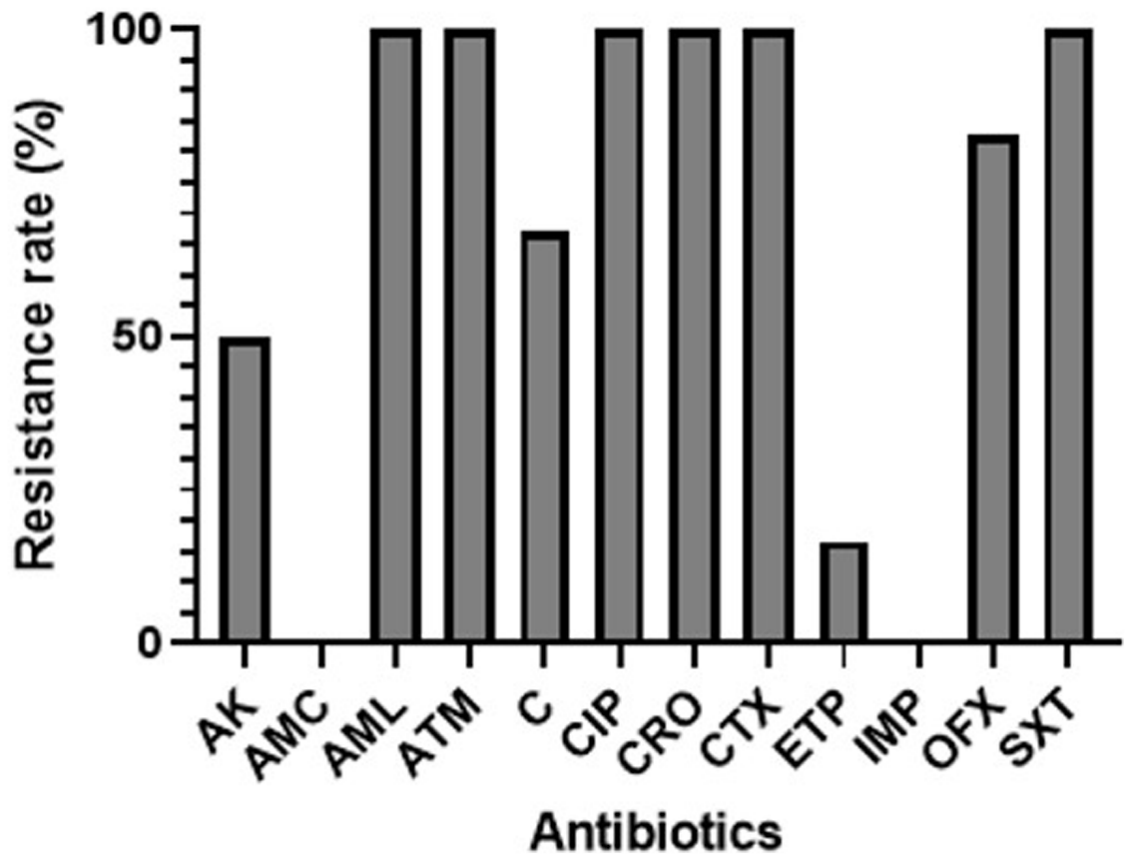
Percentage rates of antibiotic resistance among ESBL-*Ec* isolates from hospital effluents. CIP, ciprofloxacin; AML, amoxicillin; ATM, aztreonam; CTX, cefotaxime; AMC, amoxicillin-clavulanate; IMP, imipenem; ERT, ertapenem; AK, amikacin; C, chloramphenicol; OFX, ofloxacin; SXT, trimethoprim-sulfamethoxazole; CRO, ceftriaxone.

The antibiotic susceptibility rates of ESBL-producing *E. coli* isolated from hospital effluents are presented in Figure 2. The results demonstrated that all the isolates (100%) exhibited resistance to amoxicillin, aztreonam, ceftriaxone, ciprofloxacin, trimethoprim-sulfamethoxazole, ceftriaxone, and cefotaxime. A high rate of resistance was observed for ofloxacin (83%), chloramphenicol (67%), and amikacin (50%). However, only one isolate was resistant to ertapenem (16.66%), and no resistance was observed to amoxicillin-clavulanate (0%) or imipenem (0%).

### 3.4. Detection of ESBL-encoding genes

**Figure 3:**
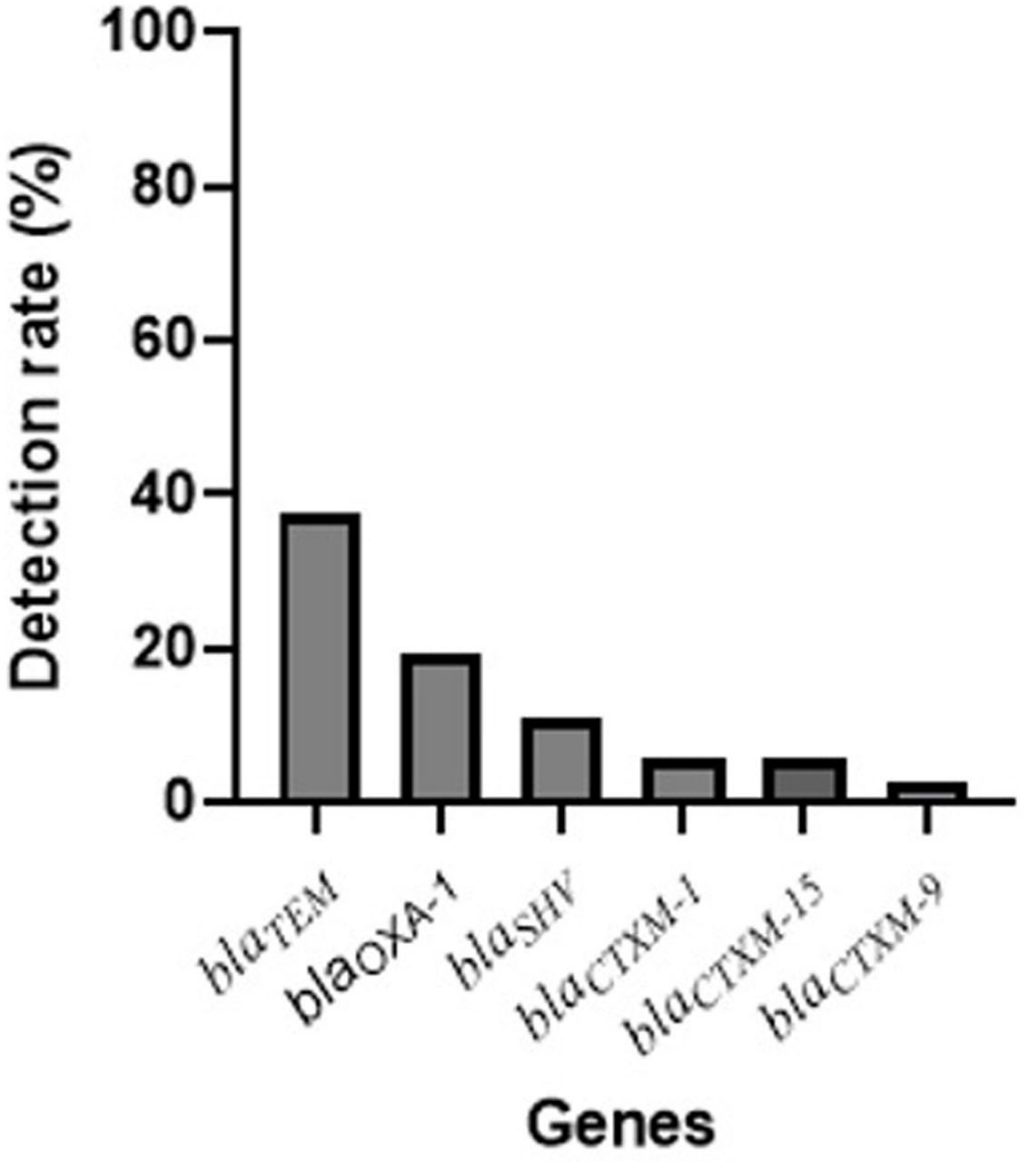
Distribution of resistance genes in ESBL-*Ec* isolates.

Figure 3 presents the distribution of antibiotic resistance genes detected in the ESBL-*Ec* isolates. The most commonly detected genes were *bla_TEM_* (37.5%), *bla*_*OXA*-1_ (19.44%) and *bla_SHV_* (11.11%). A few isolates carried bla_CTXM-1_/bla_CTXM-15_ (5.55%) and bla_CTXM-9_ (2.77%). 4.16% of ESBL-*Ec* isolates harbored three and 12.5% of ESBL-*Ec* isolates harbored two of the genes tested for.

## 4. Discussion

The Global Surveillance of ESBL-producing *E. coli* Tricycle Project, a collaborative effort between the World Health Organization (WHO), the Food and Agriculture Organization (FAO), and the World Organisation for Animal Health (OIE) (9), aims to monitor the prevalence and spread of Extended-Spectrum Beta-Lactamase (ESBL)-producing *E. coli* bacteria across humans, animals, and the environment using a “One Health” approach. In this study, modifications were made to include food sample analysis and adapt the methodology to a hospital setting. The objective of the study was to describe the prevalence of ESBL-producing *E. coli* in healthy pregnant women, the food chain, and the environment within four tertiary hospitals in Benin. A total of 296 stool samples from pregnant women were collected from four tertiary hospitals among which 22.30% (66) ESBL-producing *E. coli* isolates were detected. A study conducted in Antananarivo, Madagascar, reported quite a similar prevalence of 30% of ESBL-producing *E. coli* in healthy pregnant women (36). Two years prior, the same research team found an ESBL-producing *E. coli* prevalence of 34% in pregnant women residing in Toamasina and Ambatondrazaka (1). These high rates of ESBL prevalence place Benin among the countries with the highest rates in the global ESBL epidemiology (30). The elevated frequency of ESBL-producing *E. coli* carriage increases the risk of infections caused by ESBL producers and may necessitate the use of broad-spectrum beta-lactam antibiotics such as carbapenems, which can contribute to the propagation of carbapenem-resistant strains (1). The analysis of the variables studied here showed no significant associations (*p* > 0.05) with ESBL-*Ec* carriage in pregnant women. Previous studies have suggested that the wet season may be a factor correlated with ESBL-producing *E. coli* carriage in pregnant women (36).

The presence of ESBL-producing *E. coli* in hospital effluents is of particular concern. In this study, all *E. coli* isolates from hospital effluents were found to be ESBL producers, consistent with the findings of Markkanen et al. (21), who identified hospital effluents as hotspots for ESBL genes. Similarly, Gumede et al. (37) reported a high number of ESBL-producing *E. coli* in hospital wastewater in South Africa. The presence of *E. coli* in effluents suggests inadequate effluent management and the potential transfer of bacteria between wastewater and the surrounding environment (38). Antibiotic-resistant bacteria, including ESBL-*Ec*, found in food, pose a significant public health concern (39). However, in this study, ESBL-producing *E. coli* was not detected in food and drinking water samples. The samples primarily included ready-to-eat foods, fresh foods, and vegetables, which may explain these results.

Regarding the antibiotic susceptibility rates, the results indicated that a high number of isolates exhibited resistance to most of the tested antibiotics, including amoxicillin, aztreonam, ceftriaxone, ciprofloxacin, trimethoprim-sulfamethoxazole, ceftriaxone, and cefotaxime. However, almost all isolates were susceptible to ertapenem, amoxicillin-clavulanate, and imipenem. Similar resistance rates have been observed in other studies (1, 36). This aligns with the widely reported observation of resistance among ESBL-producing organisms against multiple antibiotics, including cephalosporins (40). Furthermore, our study investigated the presence of resistance genes associated with ESBL production. CTX-M-type ESBLs were found to be the prevailing subtype globally, with a higher incidence compared to SHV and TEM ESBLs in most geographical locations. However, in our study, the most commonly detected genes were *bla_TEM_* (37.5%), *bla_OXA-1_* (19.44%), and *bla_SHV_* (11.11%). A few isolates carried *bla_CTXM-1_*/*bla_CTXM-15_* (5.55%) and *bla_CTXM-9_* (2.77%). These findings differ from other studies that reported higher detection rate of ESBL (36, 41), indicating regional variations. One reason explaining the difference could be that other genes not included in our study, such as *bla_GES_* could be present (42). Additionally, mutations within the *bla* genes can occur, resulting in alterations to their structure or function. Such mutations may render the *bla* genes undetectable by the specific assays employed in this study (43). The presence of ESBL poses a significant challenge in managing neonatal or maternal infections, potentially leading to difficulties in finding effective treatment options.

It is important to acknowledge the limitations of this study. Firstly, ESBL-producing *E. coli* strains isolated from blood cultures, representing the human hospital component of the Tricycle project, could not be included due to the limited use of blood cultures in Benin due to the cost constraints. Secondly, the Tricycle protocol required the selection of three to five colonies per sample, which, however, may not represent the entire population of isolates. Finally, the samples were collected within a one-month period, making it challenging to determine whether the results reflect an intermittent or consistent situation. Sampling at different times of the year would provide a more reliable profile of the ESBL prevalence. Furthermore, whole genome sequencing would provide a detailed analysis of an organism’s complete genetic makeup, enabling the identification of additional resistance genes, mutations, or other genetic alterations that might contribute to the resistance phenotype (44).

This study highlights the rising prevalence of multidrug-resistant ESBL-producing *E. coli* in healthy pregnant women and the hospital environments, which raises concerns about persistent colonization in the intestines and the potential transfer of ESBL genes to other gut microorganisms. These findings contribute to the understanding of ESBL epidemiology and emphasize the need for comprehensive surveillance and intervention strategies to combat the spread of antibiotic resistance in Benin and globally.

## Data availability statement

The raw data supporting the conclusions of this article will be made available by the authors, without undue reservation.

## Ethics statement

The Ethics and Research Committee of the Institute of Applied Biomedical Sciences (CER-ISBA) reviewed and granted approval to the research proposal with the reference number 154. Prior to participating in the study, every patient or their parent/guardian provided written informed consent and received a brief explanation regarding the study’s purpose.

## Author contributions

VD, HK, BL, KH and KS wrote the protocol, did the statistical analyses, and wrote the draft of the manuscript. KF, JH, and HA processed the samples. All authors contributed to the article and approved the submitted version.

## Conflict of interest

The authors declare that the research was conducted in the absence of any commercial or financial relationships that could be construed as a potential conflict of interest.

## Publisher’s note

All claims expressed in this article are solely those of the authors and do not necessarily represent those of their affiliated organizations, or those of the publisher, the editors and the reviewers. Any product that may be evaluated in this article, or claim that may be made by its manufacturer, is not guaranteed or endorsed by the publisher.

## Supplementary material

The Supplementary material for this article showing examples of the electrophoresis gels can be found online at: https://www.frontiersin.org/articles/10.3389/fpubh.2023.1227000/full#supplementary-material
