# Supplementary material for: Prevalence and characterization of ESBL-producing Escherichia coli in healthy pregnant women and hospital environments in Benin: an approach based on Tricycle
Source: Front Public Health. 2023 Sep 28;11:1227000. doi: 10.3389/fpubh.2023.1227000 (PMC10569593; doi:10.3389/fpubh.2023.1227000)
Supplement: Supplementary file 1 [file Data_Sheet_1.pdf]

Supplementary Material

Prevalence and Characterization of ESBL-Producing Escherichia coli in Healthy Pregnant Women and Hospital Environments in Benin: an approach based on Tricycle

Kevin Sintondji (maelkevinprince@gmail.com), Kafayath Fabiyi (kafayathfabiyi@yahoo.fr), Jules Hougbenou (hougbenoubeaugard@gmail.com), Hornel Koudokpon (charleshornel@yahoo.fr), Boris Lègba (boris.legba5@gmail.com), Hornella Amoussou (amoussouhornella@gmail.com), Kaisa Haukka (kaisa.haukka@helsinki.fi), Victorien Dougnon* (victorien.dougnon@gmail.com)

*Corresponding Author: Victorien Dougnon, victorien.dougnon@gmail.com

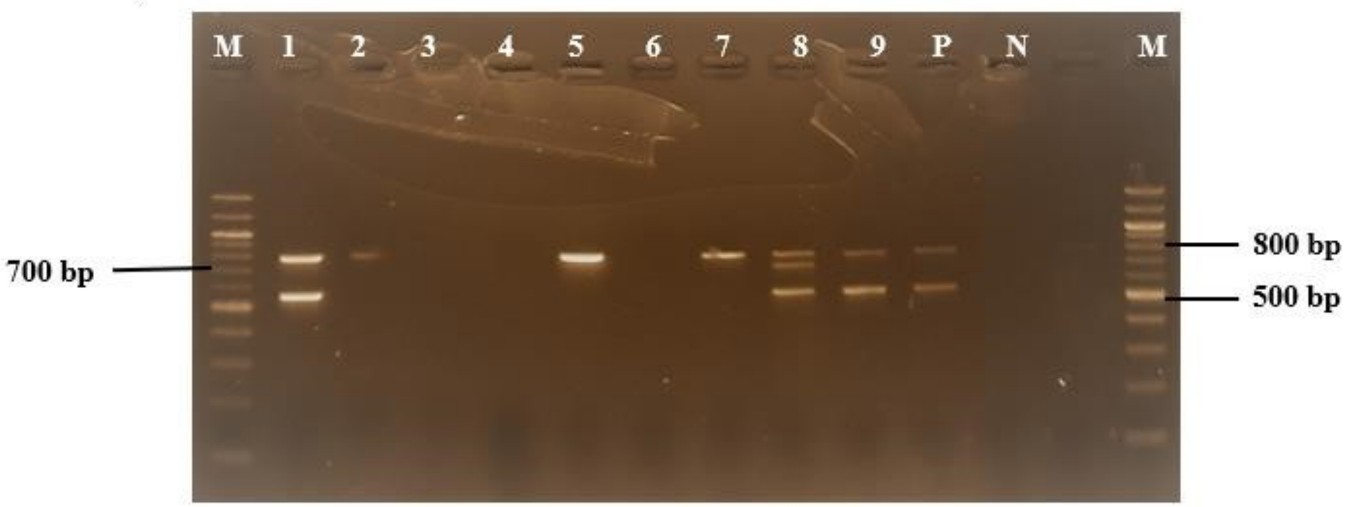

Legend: (M): Gene ruler 100pb; (N): Negative control; (P): Positive control; (8): Positive sample for bla_{SHV} gene; (1, 2, 5, 7, 8, 9): Positive samples for bla_{TEM} gene; (1, 8, 9): Positive samples for bla_{OXA-1} gene.

Figure 1: Electrophoresis profile of bla_{TEM} gene (800bp), bla_{SHV} gene (713bp), bla_{OXA-1} gene (564bp).

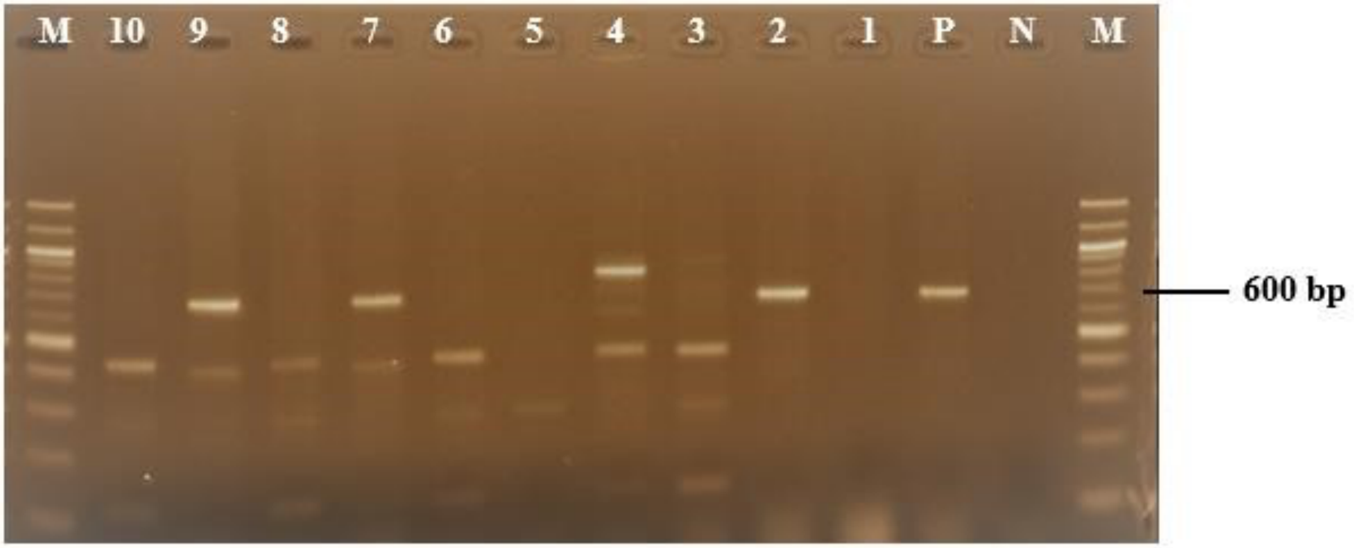

Figure 2: Electrophoresis profile of *bla_{CTXM-1}* gene (688bp)

Legend: (M)Gene ruler 100pb; (N): Negative control; (P): Positive control; (2, 7, 9): Positive samples; (1, 3, 4, 5, 6, 8): Negative samples

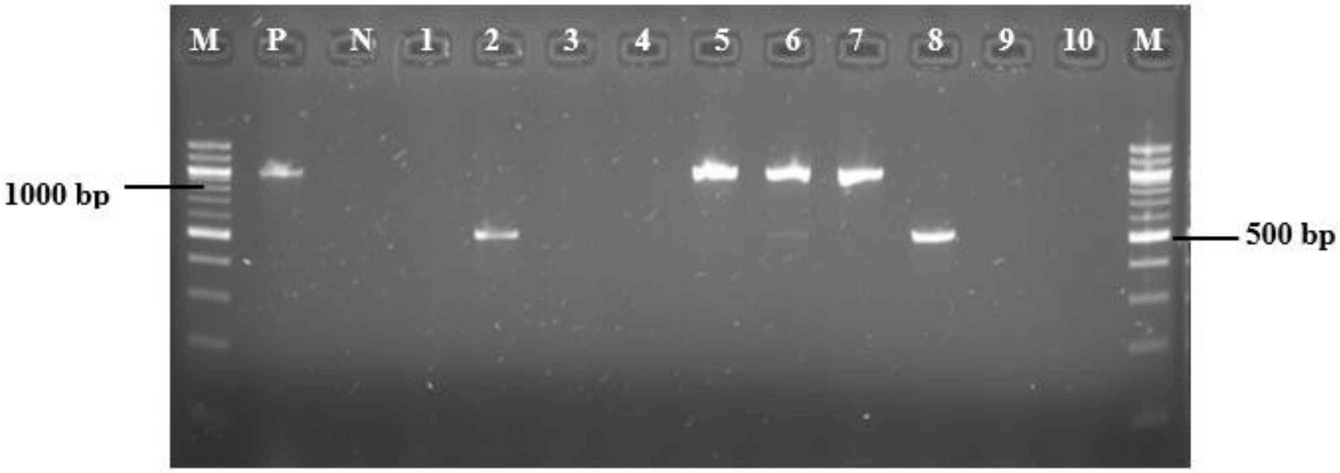

Legend: (M): Gene ruler 100pb; (N): Negative control; (P): Positive control; (5, 6, 7): Positive samples; (2, 8): Positive samples *bla_{CTXM-9}* gene

Figure 3: Electrophoresis profile of *$bla_{CTXM-15}$* gene (995bp), *bla_{CTXM-9}* gene (561bp)
